# A Rare Case of Plasmablastic Lymphoma in a Patient with HIV and SARS-CoV-2 Infections

**DOI:** 10.3390/curroncol29030129

**Published:** 2022-03-02

**Authors:** Adriana Teodora Campeanu, Elena Dumea, Mihaela Rus, Claudia Fodor, Anita Cristina Ionescu, Elena Mocanu, Mihaela Botnarciuc, Irina Magdalena Dumitru

**Affiliations:** 1 Doctoral School, “Ovidius” University of Constanta, 900470 Constanta, Romania; adrianacampeanu2003@yahoo.com (A.T.C.); cristina-ionescu@365.univ-ovidius.ro (A.C.I.); 2 Faculty of Medicine, “Ovidius” University of Constanta, 900470 Constanta, Romania; psiholog_m@yahoo.com (M.R.); elena.mocanu@univ-ovidius.ro (E.M.); mihaela.botnarciuc@univ-ovidius.ro (M.B.); dumitrui@hotmail.com (I.M.D.); 3 Clinical Infectious Diseases Hospital Constanta, 900709 Constanta, Romania; claufod@yahoo.com; 4 Faculty of Psychology, “Ovidius” University of Constanta, 900527 Constanta, Romania; 5 Oncological Institute, Prof. Dr. Alexandru Trestioreanu, 022328 Bucharest, Romania

**Keywords:** HIV, COVID-19, non-Hodgkin’s lymphoma, Epstein–Barr, oral manifestation

## Abstract

Lesions commonly associated with HIV infection include oral candidiasis, herpes simplex infection, oral Kaposi’s sarcoma, hairy leukoplakia, periodontal diseases (linear gingival erythema and necrotizing ulcerative periodontitis), xerostomia, human papillomavirus-associated warts, aphthous ulcers, non-Hodgkin’s lymphoma, histoplasmosis, carcinoma, exfoliative cheilitis, and HIV salivary gland disease. Non-Hodgkin’s lymphoma (NHL) is the most common cancer in people living with HIV (PLWH), and the incidence is increased for aggressive B-cell NHL. Plasmablastic lymphoma (PbL) is a rare and aggressive B-cell malignancy that is often unresponsive to chemotherapy and usually has a poor prognosis. We hereby present the case of a patient with a recent history of COVID-19 infection who was diagnosed with HIV and NHL, with manifestations in the oral cavity and a favorable evolution after the introduction of antiviral therapy, specific chemotherapy, and radiotherapy. Dental expertise is necessary for the appropriate management of oral manifestations of HIV infection or AIDS, and lymphoma should be included in the differential diagnosis of any oral lesions.

## 1. Introduction

Oral manifestations are common in HIV-infected patients, especially in patients with severe immunosuppression. Most often there are early signs of diagnosis that can predict progression to AIDS [1].

Depending on their etiology, they can be classified into lesions caused by various infections (viral, fungal, and bacterial), lesions that appear as manifestations of neoplasms, diseases of the salivary glands, and other lesions caused by immunodepression or drug therapies [2].

The following viruses can cause oral manifestations: the Epstein–Barr virus (causes hairy tongue leukoplakia), herpes simplex virus (causes cold sores, herpes palatine mucosa, pharynx and esophagus, gingivostomatitis), varicella zoster virus (orofacial herpes zoster), cytomegalovirus, human papillomavirus (causes focal epithelial and connective tissue hyperplasia, papillomatous, pedunculated and sessile lesions, and is located mainly on the palate, oral mucosa, and labial commissure), and poxvirus (the etiological agent for Molluscum contagiosum) [2,3]. These manifestations are correlated with severe immunosuppression, but they may also occur in individuals with immunosuppressive therapy after organ transplantation or following systemic corticosteroid therapy [3]. In HIV patients, they are often recurrent, long-lasting, and resistant to treatment, and may also occur in immune reconstitution inflammatory syndrome [3].

Oral, pharyngeal, or esophageal candidiasis are the most common fungal infections observed as initial manifestations of HIV infection and usually have one of four clinical presentations: erythematous candidiasis, pseudomembranous candidiasis, angular cheilitis, and hyperplastic or chronic candidiasis [4]. The disease usually occurs in patients with a CD4 count of less than 300 cells/µL. The most common candida species involved is Candida albicans, but non-albicans species can also be found [4].

The most common oral lesions associated with bacterial infection are linear erythematous gingivitis and necrotizing ulcerative periodontitis. Less commonly associated lesions are bacillary epithelioid angiomatosis, syphilis, and ulcerative-necrotic gingivitis. Thus, clinical lesion is a manifestation of an altered immune response to pathogens [5].

Malignant lesions include Kaposi’s sarcoma and non-Hodgkin’s lymphoma (NHL). Kaposi’s sarcoma is the most common intraoral malignancy associated with HIV infection [6,7].

Non-Hodgkin’s lymphoma appears as a rapidly growing mass, not similar to an ulcer or plaque, and most commonly appears on the palate or gingiva. A histological examination is essential for diagnosis and staging [8]. The prognosis is poor, with a median survival of less than 1 year, despite chemotherapy [9].

Oral lesions as well as manifestations of lymphomas associated with HIV infection are less common but should be considered, especially in patients with advanced HIV infection who are at higher risk of developing these conditions [10]. In these patients, the risk of developing non-Hodgkin’s lymphoma is 100–200 times higher compared to that in the general population [11], and this malignancy ranks second, after Kaposi’s sarcoma [12].

## 2. Case Presentation

**Figure 1:**
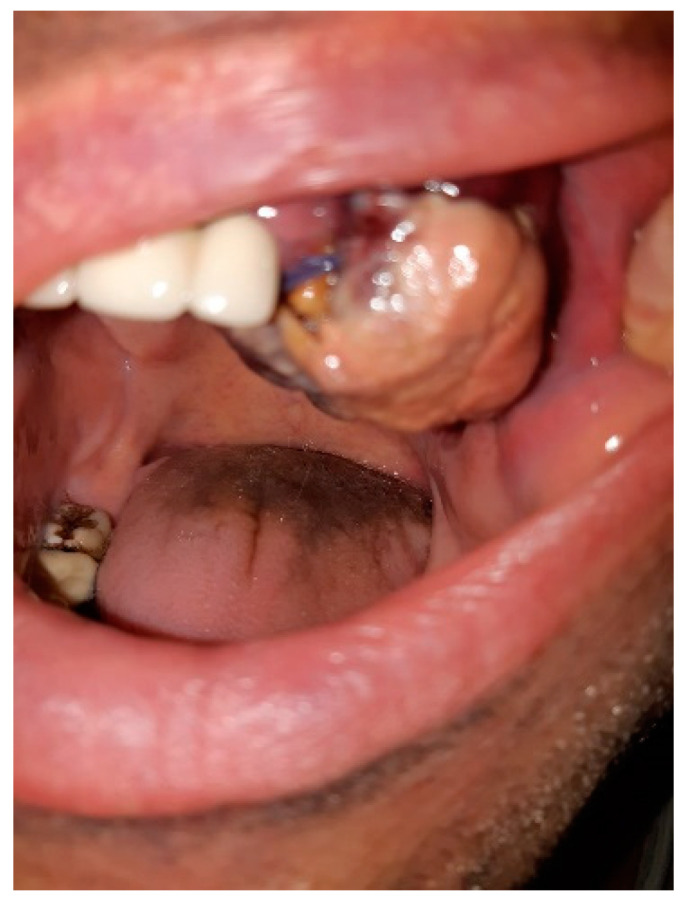
Ulcer-vegetative tumor lesion in the oral cavity.

We hereby present the case of a 51-year-old patient, a smoker, with a history of severe SARS-CoV-2 infection in October 2020, who developed an ulcer-vegetative tumor lesion in their oral cavity in November 2020 (one month after discharge). The lesion was initially located on the left mandibular level (alveolar ridge) and later extended to the upper dental arch, vestibule, and palate, with a rapid growth in volume (Figure 1). Symptoms included profuse sweating, fatigue, loss of appetite, and weight loss (at approximately 7 kg in the last month).

Laboratory tests showed anemia (Hb: 10.2 g/L, *n* = 12.6–17.4 g/L), leukopenia with lymphopenia (WBC: 4.1 × 10^9^/L, *n* = 4–10 × 10^9^/L; Ly: 1.1 × 10^9^/L, *n* = 1–4 × 10^9^/L), modified inflammatory samples (ESR: 58 mm/h, *n* = 3–9 mm/h; Fb: 474 mg/dl, *n* = 196–372 mg/dl; CRP: 0.970 mg/L, *n* = 0–5 mg/L), serological markers for hepatitis B and C negative (AgHBs: negative; anti-HBc: negative; Ac anti-HCV: negative), positive HIV test, IgG Epstein–Barr positive, and EBV DNA titers = 213,000 copies/mL.

From their recent history, we noted that the patient had been hospitalized for 18 days in a COVID department and received treatment with remdesivir (5 days), tocilizumab, and dexamethasone of 16 and then 8 mg/day, for a total of 14 days, with a favorable evolution; at discharge, he showed normal blood counts and inflammatory samples and a PCR that was SARS-CoV-2 negative.

Cranial computed tomography (CT) evaluation described numerous osteolytic lesions in the skull cap, the largest at 17/10 mm in the left parietal. Pulmonary CT showed areas in matte glass (COVID sequelae lesions) and bilateral axillary lymphadenopathy with diameters of 23.4/16.6 mm, without mediastinal lymphadenopathy, and an abdominal/pelvic CT showed only small juxta-centimetric lymphadenopathy located in the lower cervical celio-mesenteric and intra-mesenteric.

HIV RNA of 965,584 copies/mL and a low value of CD4 of 197 cells/μL (*n* = 300–1400 cells/μL) confirmed the diagnosis of HIV infection and probably Kaposi’s sarcoma with oral manifestations (CD8: 1137 cells/μL, *n* = 200–900 cells/μL; CD4/CD8: 0.17, *n* 1–3.6) and antiretroviral therapy was given with genvoya at 150/150/200/10 mg (elvitegravir/cobicistat/emtricitabine/tenofovir alafenamide) via one tablet per day with food, a treatment that the patient tolerated and to which he was adherent.

Tumor resection and histopathological examination and immunohistochemical examination were also used.

**Figure 2:**
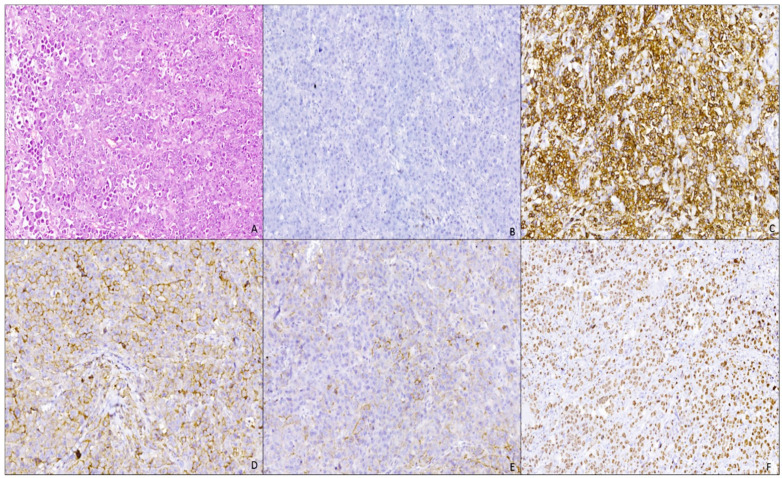
Plasmablastic lymphoma of the oral mucosa: (**A**) proliferation of large atypical cells with immunoblastic features (H&E stain, Ob. ×100); (**B**) negative immunostain for CD20 antibody (IHC, Ob. ×100); (**C**) intense and diffuse membranous immunostaining for CD38 antibody (IHC, Ob. ×100); (**D**) moderate and diffuse membranous and cytoplasmic immunostaining for Kappa antibody (IHC, Ob. ×100); (**E**) moderate and focal membranous and cytoplasmic immunostaining for lambda antibody (IHC, Ob. ×100); (**F**) intense and diffuse nuclear immunostaining for KI67 antibody, with a high KI67 index of −90% (IHC, Ob. ×100).

Histopathological examination in hematoxylin and eosin (H&E) staining showed multiple tissue fragments with diffuse malignant lymphoid tumor proliferation areas with monomorphic large cell rounded nuclei, with either eccentric or centrally localized nuclei, vesicular chromatin, prominent nucleolus, frequent atypical mitosis, and with abundant or moderate eosinophilic cytoplasm, displaying an immunoblastic picture (Figure 2A). The malignant cells were arranged in cohesive sheets with focal areas of tumoral necrosis.

Four-micromillimeter-thick sections from formalin-fixed, paraffin-embedded tissue were used for immunohistochemistry performed by an automated staining system (Ventana BenchMark GX Automated System). The following primary antibodies were used after antigen retrieval in citrate buffer: AE1/AE3; cluster designation (CD)20; CD3; CD38; CD138/syndecan-1; CD56; PAX5; anaplastic lymphoma kinase (ALK1); KI67; anti-kappa; anti-lambda.

Immunohistochemical examination showed that tumor proliferation was negative for CD20 (Figure 2B) and PAX5 (B cell marker); negative for CD3 (T-cell marker); diffuse positive for CD38 (Figure 2C) and CD138 (plasma markers); negative immunoreaction for CD56 (NK marker, positive aberrant in 80% of cases of plasmacytoma or multiple myeloma); negative immunoreaction for AE1/AE3 (anti-pan keratin). The proliferation exhibited a clonal character with a kappa/lambda ratio of 10:1 (Figure 2D,E) and a high Ki67 proliferation index (Figure 2F). A negative immunoreaction was also noted for ALK1 (anaplastic lymphoma kinase).

The histopathological appearance and immunohistochemical staining (positive reaction specific for a plasma cell phenotype) indicated a diffuse plasmablastic proliferation with the secretion of light kappa-type chains. These results, correlated with clinical data and alveolar localization, and with no reaction to AE1/AE3, CD56, CD20, and PAX5 antibodies, support the diagnosis of plasmablastic lymphoma (determination of the left alveolar ridge) with HIV infection, stage B3 (CDC classification).

The patient was evaluated in February 2021 after 2 months of antiviral therapy and considering the favorable evolution (HIV RNA: 272 copies/mL; CD4: 439 cells/μL; CD8: 1510 cells/μL; CD4/CD8: 0.29) of the initiation of chemotherapy treatment in a hematology department. Bone marrow aspiration was performed and revealed cellularity with a relatively normal density and granulocyte series reduced in percentage, with a slight deviation to the left to myeloblasts of 3–4%, approximately 6–7% for lymphocytes, and 10–12% for plasma cells (without affecting the disease).

For dose-adjusted-EPOCH chemotherapy (etoposide phosphate, prednisone, vincristine sulfate (Oncovin), cyclophosphamide, and hydroxydaunorubicin), six chemotherapy cures that were relatively well tolerated and without significant hematological side effects were initiated. The positron emission tomography–computed tomography (PET–CT) performed in June 2021 detected metabolically active lesions at the level of alveolar recess, left maxillary sinus, and moderately captured infracentimetric lymphadenopathy located on the left submandibular and perijugular radiotracer. Radiotherapy was used between July and August 2021 with a total dose of external radiation of 44 Gy (20 fractions × 2.2 Gy, 5 days per week, conventional fractionation) using the IMRT-VMAT technique (intensity-modulated radiotherapy combined with volumetric modulated-arc therapy). The subsequent PET–CT evaluation showed a favorable evolution 9 months after diagnosis, but should be mentioned that PBL cases are reported to relapse within a median of 6 months, despite often achieving complete remission during first-line treatment (Table 1).

## 3. Discussions

Non-Hodgkin’s lymphomas (NHLs) remain a major cause of morbidity and mortality for HIV-infected patients, and treatment outcomes are influenced by chemotherapeutic regimens and effective antiretroviral therapy [13], as well as the management of side effects and drug interactions.

Oral manifestations in the NHL are second in frequency (11–33%) after gastrointestinal damage [12]. Plasmablastic lymphoma (PbL) is a rare and aggressive B-cell malignancy that is often unresponsive to chemotherapy and usually has a poor prognosis [14]. The association with HIV infection is often described, but it is also noted in immunocompetent individuals and post-transplant patients [14]. In HIV-infected patients, EBV infection causes atypical lymphoproliferations and malignant transformations of lymphoid cells through a combination of mechanisms, including the use of virus-encoded transforming genes, the stimulation of diverse cytokines, and interaction with receptors for the tumor necrosis factor (TNF) [15]. Ting Chen et al., in a study conducted in 2019 at the beginning of the COVID-19 pandemic, reported the presence of IgM EBV in several patients with COVID 19. In these patients, more severe forms of the disease were more common, with higher CRP values, and corticosteroids being used more frequently [16]. bL is usually associated with Epstein–Barr virus infections, with more than 80% of HIV-positive cases and PbL presenting positive serologies [17], and immunohistochemical analysis is crucial for confirmation [18]. In addition, in people with HIV infection, PbL can occur even in conditions of acceptable immunity in people undergoing antiretroviral therapy and having a CD4 greater than 200 cells/mm^3^ [19].

Achenbach CJ et al., in a 2014 study of 18,382 HIV-infected patients, both naive in antiretroviral therapy and effective ARV therapy, showed an increased incidence of NHL even in patients with CD4 counts of >200 cells/mm^3^ and suggested a role of HIV viremia in the pathogenesis of NHL [20]. Other studies show that high-level HIV viremia (>10,000 copies/mL) and cumulative HIV viremia are predictive of NHL independent of nadir and the time variance of CD4, starting from the finding that viral replication causes immune dysfunction and B-cell activation, which increases the risk of NHL [21].

The prognosis for patients with PbL is generally poor, and the average survival rate is 6–9 months [14]. Patients with localized disease have a better prognosis; also, several studies conducted in HIV-infected patients diagnosed with PbL have shown that a favorable outcome is more common in HIV-positive patients, and the administration of antiretroviral therapy plays a particularly important role [14]. In our case, potent antiretroviral therapy was administered early, concomitantly with chemotherapy and followed by radiotherapy, with a favorable outcome: an HIVRNA of <200 copies/mL, with PETT–CT not detecting pathological changes 9 months after diagnosis.

Regarding SARS-CoV-2 infection, recent studies suggest that it has a pro-oncogenic effect, either directly by blocking the autophagic flux and leading to immune escape by the downregulation of major histocompatibility complex I (MHC-I) or indirectly through the possible reactivation of Epstein–Barr virus [22,23]. However, numerous authors have reported that there is a significant decrease in peripheral lymphocytes and natural killer (NK) cells in patients with COVID-19 [24]. Pasin F et al. reported, in a 20-year-old patient, the transient remission of refractory NK/T-cell lymphoma during COVID-19 infection, suggesting that SARS-CoV-2 may have an oncolytic effect [25]. Other authors report similar cases of the favorable evolution of hematological cancers or solid tumors during SARS-CoV-2 infection, but the number of such reports is very low, and no conclusion can be drawn [26,27].

In our case, the factors that could have influenced the appearance and evolution of the NHL were HIV infection with increased viremia, the presence of Epstein–Barr virus, and possibly infection with SARS-CoV-2.

## 4. Conclusions

Oncological expertise is necessary for the appropriate management of oral manifestations of HIV infection or AIDS, and lymphoma should be included in the differential diagnosis of any oral lesions.

Immunohistochemistry and correlation with clinical findings are crucial for establishing a correct diagnosis.

The favorable evolution of PbL was influenced by the early administration of effective chemotherapy as well as the early introduction of highly active antiretroviral therapy.

The role of SARS-CoV-2 infection in the evolution of cancers seems interesting and deserves to be studied in larger groups of patients.

## Figures and Tables

**Table 1:** Patient’s timeline.

October 2020	November 2020	December 2020	February 2021	July–August 2021	September 2021
COVID-19	Ulcer-vegetative tumor lesion in the oral cavity HIV	Plasmablastic lymphoma Start ART (genvoya)	Dose-adjusted-EPOCH chemotherapy (six cures)	Radiotherapy	PETT–CT favorable evolution
	CD4: 197 cells/μL; CD8: 1137 cells/μL; CD4/CD8: 0.17		CD4: 439 cells/μL; CD8: 1510 cells/μL; CD4/CD8: 0.29		CD4: 542 cells/μL; CD8: 1420 cells/μL; CD4/CD8: 0.38
	HIVRNA: 965,584 copies/mL		HIVRNA: 272 copies/mL		HIVRNA < 200 copies/mL

## Data Availability

All data generated or analyzed during this study are included in this published article.
